# Ultrasensitive detection of DNA and protein markers in cancer cells

**DOI:** 10.7497/j.issn.2095-3941.2015.0048

**Published:** 2015-09

**Authors:** Irina V. Smolina, Natalia E. Broude

**Affiliations:** Department of Biomedical Engineering, Boston University, Boston, MA 02215, USA

**Keywords:** Microfluidic droplets, rolling circle amplification (RCA), peptide nucleic acids (PNA), cell-surface protein marker

## Abstract

Cancer cells differ from normal cells in various parameters, and these differences are caused by genomic mutations and consequential altered gene expression. The genetic and functional heterogeneity of tumor cells is a major challenge in cancer research, detection, and effective treatment. As such, the use of diagnostic methods is important to reveal this heterogeneity at the single-cell level. Droplet microfluidic devices are effective tools that provide exceptional sensitivity for analyzing single cells and molecules. In this review, we highlight two novel methods that employ droplet microfluidics for ultra-sensitive detection of nucleic acids and protein markers in cancer cells. We also discuss the future practical applications of these methods.

## Introduction

Cancer cells differ from normal cells in various parameters, which define the uncontrolled growth, lack of cell-cell communication, and loss of adhesive properties of cancer cells. All these variations are caused by genomic mutations and altered gene expression. Early detection of cancerous changes is important because it increases the probability of treating the disease and even reversing malfunction in cells and tissues. For practical applications, diagnostic methods should satisfy several important criteria: specificity and low false-positive rates; sensitivity and low false-negative rates; simplicity and lack of the need to use sophisticated equipment to be applicable in field conditions; and rapidity in delivering results. Current available assays only reveal key information, such as molecular heterogeneity, functional variation, and variability of drug-target interactions, at the single-cell and single-molecule levels. In this regard, recent studies have focused on the development of effective technologies, such as flow cytometry and single-cell fluorescence spectroscopy, for examining complex biological processes in cancer cells at the single-molecule or single-cell level.

**Figure 1:**
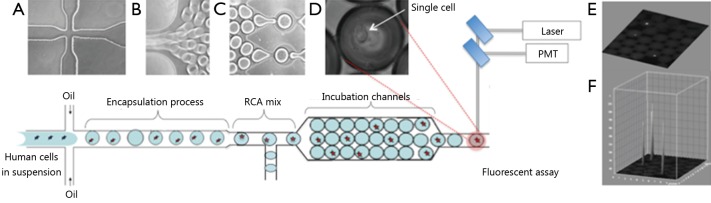
Design of microfluidic nanoliter platform for gene-specific cell identification. (A) Schematic top view of the cross-section for generation of monodisperse aqueous droplets; (B) Droplets are conveyed by the oil focused in the microfluidic channel; (C) Chip-integrated RCA reaction module that enables on-chip incubation of the droplet for 30 min; (D) Phase-contrast image of single-cell encapsulated in the droplet; (E) Fluorescence image of encapsulated cells within a droplet array; (F) Quantitation plot of the 3D surface of fluorescence intensity distribution in (E).

Droplet microfluidic devices have recently emerged as an effective tool used to encapsulate single cells within monodisperse microdroplets for high-throughput analysis with exceptional sensitivity. These devices utilize two immiscible fluids in microfluidic channels to rapidly create monodisperse water-in-oil nanospheres, called droplets, which then function as individual reaction vessels, with volumes ranging from a few femtoliters (fL) to nanoliters (nL)^1^. For single-cell studies, droplets should contain at most one cell so that the majority of drops do not contain cells at all, given that the encapsulation process follows Poisson statistics. A previously described polydimethylsiloxane microfluidic system generates monodisperse droplets in a microchannel by shearing flow at a T-junction or a flow-focusing zone^2^^-^^4^. This microfluidics platform is schematically illustrated in **Figure 1**. The system contains three perpendicular inlet channels, which form a nozzle. The center stream comprises the suspension containing cells (~10^5^ cells/mL), and the two opposing side streams contain the oil phase. Individual syringe pumps control the flow rate of oil and cell suspension. The droplets generated can be manipulated for mixing, merging, sorting, thermocycling, and fluorescence detection (by using photomultiplier tubes or avalanche photodiodes), which are procedures required for particular assay protocols^5^^,^^6^. The features of droplet microfluidic systems not only permit high-throughput analysis using minimal amounts of reagents but also reduce solute-surface interactions and cross-contamination of the reagents. Droplet microfluidic systems can also be applied to characterize and isolate individual cells for further analysis of genetic materials and proteins.

**Figure 2:**
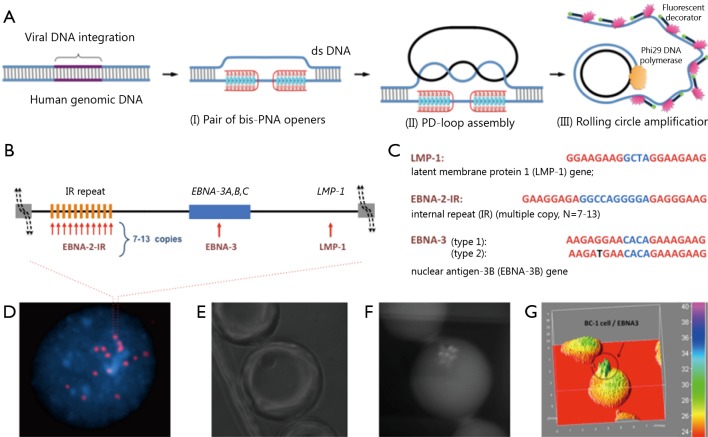
PNA-RCA method for detection of short DNA target sites at the single-cell level. (A) (I) PNA openers specifically bind to two closely located homopurine DNA sites, which are separated by several random purine-pyrimidine bases, and locally open the double-stranded DNA; (II) The opened region serves as a target for hybridization and ligation of an oligonucleotide probe to form a PD-loop; (III) The small circle on duplex DNA serves as a template for isothermal RCA reaction, which yields a long, single-stranded amplicon that contains thousands of copies of the target sequence. For the fluorescence-based detection, fluorophore-tagged decorator probes are hybridized to the RCA product. (B) A map of viral genomic locus used for viral identification. (C) Target sites in the EBV genomic DNA used as genetic biomarkers. Sequences targeted by PNAs are shown in red. EBNA-3(G)-EBV type 1 signature sites within the EBNA-3 gene differ by a single nucleotide (SNP) from EBNA-3(T)-EBV type 2 in the PNA binding sequence. Mismatch is shown in black. (D) Multiple fluorescent spots were detected by fluorescent microscopy in BC-1 cells (EBV-positive) upon application of the probe corresponding to the LMP-1 gene encoding major transforming protein of EBV. The fluorescent signals were acquired separately using two filter sets, namely, DAPI for DNA and Cy3 for labeled RCA product; (E-G) For quantitation of oncoviral DNA target sites within BC-1 cells, fluorescence intensities were recorded for the single-copy genes LMP-1 and EBNA-3 and for multiple copies of the EBNA-2 IR target sites. Each droplet contains a single cell. Representative droplet images: (E) DIC; (F) CY3; (G) The fluorescence intensities from these images are plotted as 3D surface graphs using ImageJ (top panel, the color code at the right indicates fluorescence intensities).

**Figure 3:**
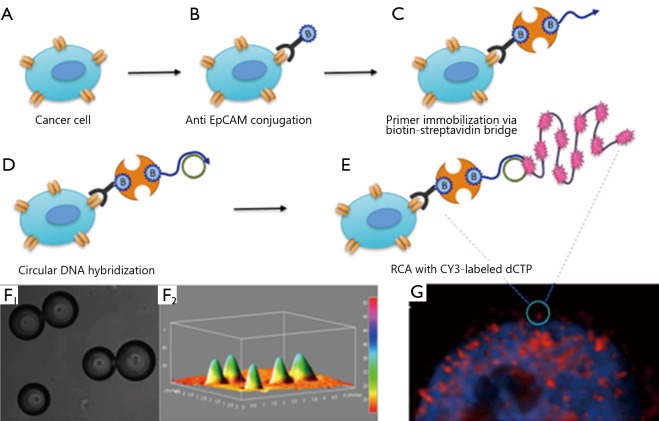
Schematic illustration of the assembly of the complex for detection of marker surface proteins. (A) Cancer cell. (B) Binding of biotin-labeled antibodies. (C) Coupling of biotinylated DNA-tag primer via streptavidin bridge. (D) Hybridization of the DNA minicircle. (E) RCA in the presence of CY-3-labeled dCTP. (F_1_) A fluorescence image of encapsulated cells within a droplets array. (F_2_) 3D surface plot of the fluorescence-intensity distribution in F_1_. (G) Microscopy images of a PC3 cell fixed on the glass slide. Expression of the EpCAM marker (red patches) detected via the conjugated RCA strategy, wherein cells were counterstained with 4,6-diamidino-2-phenylindole (DAPI, blue).

In this review, we highlight two novel methods for detection of DNA mutations and protein markers in cancer cells and discuss the advantages and limitations of each approach for practical applications (**Figures 2****,****3**). Both methods involve rolling circle amplification (RCA), which allows isothermal signal amplification. RCA is a simple amplification method that features single-molecule sensitivity. This method also exhibits high signal amplification efficiency and sequence specificity, which are attributed to the padlock probe design and DNA ligase activity that requires perfect DNA complementarity under stringent conditions^7^. RCA is an isothermal reaction and therefore does not require thermal cycling, which entails sophisticated and expensive instrumentation, such as in microfluidic droplet systems integrated with polymerase chain reaction. Assuming that RCA replication rate using phi29 DNA polymerase is about 1,500 bases per min^8^, we can estimate that each rolling-circle product (RCA amplicon) induces the accumulation of ~90,000 nucleotides per hour, which appear as a fluorescent signal on every cell. The signal is recorded and quantified for each cell in the droplet through comparison with empty droplets and thus represents the background signal. **Figure 1** shows an interactive 3D surface plot of fluorescence intensity distribution (vertical z axis) within each droplet at the end of amplification. In this setting, real-time RCA in nanoliter drops can provide quantitative measurements if fluorescence is recorded using a custom optics system and software^3^.

## Genetic marker detection

Detection and analysis of nucleic acids are crucial for cancer research, diagnostics, and therapy. Analysis at the single-molecule level is important because it elucidates the genomic characteristics of each molecule and facilitates the identification of abnormal genes. Variations in the human genome, which range from chromosomal alterations involving millions of base pairs to single-nucleotide polymorphisms (SNPs), can lead to the development of cancer. As such, scholars have contributed tremendous efforts to develop molecular technologies for analysis of allelic variations. Methods used to analyze genomic mutations vary depending on the scale of changes, which cover the whole spectrum of methods with one extreme as total genome sequencing to reveal single SNPs up to another extreme, namely, chromosome analysis, to determine chromosome rearrangements involving millions of base pairs^9^. A novel method has been recently described for ultrasensitive detection and quantitation of short DNA mutations in single cells with single-base resolution. This method helped to fill the void in methodological approaches: it can be applied to the entire human genome and at the same time it has a single base resolution. In other words it works as DNA fluorescence *in situ* hybridization (FISH) but opposite to FISH has an extremely high resolution^10^. The method utilizes two molecular tools, namely, peptide nucleic acids (PNAs) and RCA.

DNA-mimicking PNAs are a convenient tool for research and diagnostic assays^11^^,^^12^. PNAs are a prominent class of artificial nucleic acid analogs with peptide-like backbones, onto which nucleobases are grafted in a designed sequence^13^. Cationic pyrimidine bis-PNAs exhibit strong and selective binding to duplex DNA, thereby creating a local opening in a selected genomic target site. PNA binds to one DNA strand and renders another strand to be available for hybridization. The displaced DNA strand becomes accessible for Watson-Crick pairing with a DNA oligomer to form an unusual PNA-DNA construct, known as a PD-loop structure^14^. The formation of the loop is highly sequence-specific because of the simultaneous construction of Watson-Crick and Hoogsteen base pairs^15^. The PD-loop is also limited to pre-selected target sites of 20 to 25-bp in length, whereas the remaining DNA preserves its double-helical structure. Moreover, the PD-loop construct can be used for amplification and fluorescence detection of various genetic markers for diagnostics (**Figure 2**). Overall, PNA-RCA method allows complete analysis at physiological temperatures and does not require isolation of genomic DNA, a step required in other DNA analyses. Thus, different assay formats can be employed to perform the analysis. The PNA-RCA approach can also be used to detect fluorescent *in situ* of short single-copy DNA sequences for bacteria identification^16^^,^^17^ and DNA genotyping in human cells^10^.

PNA-RCA method has been successfully applied in human cells to detect and identify herpesvirus, which carry oncoviral DNA inserts within their genomes^18^. Herpesviruses comprise a family of viruses that cause latent recurring infections. More than 90% of adults have been infected with at least one virus strain from this group; in most people, a latent form of the virus remains because it is incorporated into the human genome. Chromosomal integration of genetic material from herpesviruses may lead to genetic abnormalities, which cause malignant transformation^19^^,^^20^. As such, detecting herpesviruses in patients with lymphoid diseases and related disorders is clinically important because these illnesses are treated differently from morphologically similar but non-oncovirus-associated malignancies. Epstein-Barr virus (EBV), also called human herpesvirus 4, is one of the eight viruses in the herpes family and a common virus that infects humans. The pathogenesis of EBV-positive lymphomas in humans varies depending on their classification as EBV type 1 or 2^21^^,^^22^. EBV type 2 infections are less likely to cause tumors and if developed, the tumors exhibit longer incubation period compared with EBV type 1 tumors^23^. As EBV types 1 and 2 are very homologous, the differences in their viral genomes with polymorphisms in the latent genes EBNA-2, 3A, 3B, and 3C can be used for identification^24^. In our previous study, we classified EBV via the PNA-RCA method using the signature sites within the *EBNA-3* gene, which contains single G/T mutation for type 1/type 2 (**Figures 2****,****3**)^18^. Droplet-based microfluidic system was then used for PNA invasion, followed by RCA, to detect and quantify viral genomes in human cells. The results showed successful quantitative detection of the selected genomic biomarkers within single cells. Compared with that for the EBNA-2 IR repeat inserts, the fluorescence intensity for the single-copy genes LMP-1 and EBNA-3 was seven times lower, which correlates well with the known copy number within the range of 7 to 13 of the repeat, thereby indicating accurate quantitative analysis^18^. Therefore, this assay could be applied to identify both the type and multiplicity of viral infection in human cells.

## Protein marker detection

Protein analysis of cell-specific surface markers is another important approach for diagnostic and therapeutic modalities in cancer management^25^. Cell surface membrane proteins are involved in central processes, such as cell signaling, cell-cell interactions, and ion and solute transport; these proteins also exhibit pivotal function in several steps of metastatic processes. The low abundance of cell surface membrane proteins complicates their identification, especially at early stages of the illness^2^^,^^26^. Most current strategies for detection of protein markers utilize fluorescent dye-coupled antibodies, followed by flow cytometry analysis. Nevertheless, this technique presents inadequate sensitivity and can only be used for investigating highly and moderately expressed biomarkers; moreover, this method requires a minimum of several hundreds to thousands of proteins per cell to achieve a measurable signal. Detection of rare markers necessitates an amplification step based on enzymatic reaction with subsequent staining or by increasing the number of fluorophore molecules through utilization of quantum dots (Qdots), fluorescent microspheres, or extra ‘‘layers’’ of reagents^27^^,^^28^. These amplification methods include multiple steps of binding and washing of excess reagents, thereby requiring long processing time and large volumes of reagents and samples. In addition, these methods are prone to false-positive events caused by non-specific antibody binding. Thus, alternative amplification methods for protein markers must be developed for diagnostic and therapeutic applications in cancer biology and diagnostics.

We previously developed a robust method for detection of low-abundance cell-surface protein markers on single cancer cells (**Figure 3**). This method employs highly specific antigen–antibody recognition combined with DNA-amplification using RCA in a nanoliter droplet microfluidics platform. As direct chemical amplification of protein markers is not feasible, cell-surface proteins are labeled with specific biotinylated antibodies, which are later conjugated to DNA tags via a biotin-streptavidin bridge^3^ (**Figure 3**). The conjugated constructs function as scaffolds for the assembly of RCA on cell-specific markers^4^^,^^5^ (**Figure 3**). Moreover, the RCA process is isothermal; therefore, the integrity of the antibody-antigen complexes and the viability of cells are preserved^29^.

Circulating tumor cells are an excellent source of surface markers for monitoring progress in the treatment of cancer patients. An epithelial cell-adhesion molecule (EpCAM) is a transmembrane glycoprotein that mediates Ca^2+^-independent homotypic cell-cell adhesion in epithelia. EpCAM shows oncogenic potential via the capacity to up-regulate c-myc, e-fabp, and cyclins A and E. EpCAM is exclusively expressed in epithelium and epithelia-derived neoplasms and therefore can be used as a diagnostic marker for various cancers^30^. However, the expression levels of EpCAM in some tumors are lower than the threshold for direct detection using flow cytometry. In our study, we used EpCAM markers on the PC3 human prostate cancer cell line as a model system. PC3 cells were first treated with biotinylated antibodies specific for EpCAM epitope, followed by a specific DNA tag primer via the streptavidin-biotin bridge. A DNA tag primer was then used to circularize a padlock probe. The obtained minicircles were then isothermally amplified via RCA using phi29 DNA polymerase in the presence of CY-3-labeled dCTP. The linear RCA reaction yielded single-stranded DNA products, which fold into a random coil; these products appear as localized fluorescent clusters tethered to the cell surface and are clearly visible as bright “dots” under a standard fluorescence microscope. These findings demonstrated that RCA amplification provides clearly distinguishable fluorescent signals that enable robust analysis of EpCAM biomarkers on PC3 cells^7^ (**Figure 3**). By contrast, no fluorescent signals were observed in control cells (i.e., lymphocytes), which did not express EpCAM and were treated in a similar manner to PC3 cells. Hence, the assay is highly specific and does not produce false-positive results. We then demonstrated a linear dynamic response in the total fluorescence intensity within the concentration range of 1 to 10^4^ molecules/mm^2^ surface-immobilized DNA-rolling-circle. The calculations showed that the described method allows visual detection of 1 to 10 surface protein molecules per cell and amplified the signal by at least 1,000-fold compared with conventional labeling procedures^29^. Furthermore, we identified the specific tumor marker, EpCAM, on the tumor-cell surface through the microfluidic technology using miniaturized nanoliter reaction droplets.

Various therapeutic strategies could benefit from sensitive and rapid detection of surface proteins; these strategies include antibody- and gene-directed therapy, which utilize the selective expression of markers on the surface of tumor-associated cells. For example, such analysis can be performed on tumor cells before and after treatment with various therapeutic regimens. The proposed approach is highly beneficial for detection and isolation of circulating tumor cells, which can be recovered from the drops for future testing. In summary, the development of two ultra-sensitive methods, namely, PNA and RCA, for analysis of genomic and proteomic content of cells is a significant step in the advancement of cancer diagnostics. These methods use microfluidic devices to allow single-cell analysis, that is, each compartment acquires only a single cell. Both methods also employ RCA for signal amplification to allow isothermal analysis and cell integrity preservation. The DNA analysis of unique sites in single cells automatically implies a single-molecule level of detection. Moreover, data show single-base resolution can be achieved by discriminating EBV sub-types. Protein marker detection can be used to detect low-abundance markers. Nevertheless, the two methods exhibit certain limitations. Genomic analysis requires detailed knowledge of the sequence prior to selecting target sites that differ between normal and cancer cells. For proteomic analyses, antibodies for each specific marker are also needed. Furthermore, droplet microfluidic setup is not widely used yet but the current trend shows that these nanodevices will become a part of routine instrumentation in cancer research in the future.

## Conclusion

PNA and RCA methods are ideal for various applications involving selective screening of single mammalian cells by analyzing genomic and proteomic characteristics of individual cells. The combination of these methods can provide a comprehensive analysis of cancer heterogeneity at the single cell level in future applications.
